# Pharmacologic Inhibition of ADAM10 Attenuates Brain Tissue Loss, Axonal Injury and Pro-inflammatory Gene Expression Following Traumatic Brain Injury in Mice

**DOI:** 10.3389/fcell.2021.661462

**Published:** 2021-03-15

**Authors:** Dominik Appel, Regina Hummel, Martin Weidemeier, Kristina Endres, Christina Gölz, Michael K. E. Schäfer

**Affiliations:** ^1^Department of Anesthesiology, University Medical Center, Johannes Gutenberg-University, Mainz, Germany; ^2^Focus Program Translational Neurosciences (FTN) of the Johannes Gutenberg-University, Mainz, Germany; ^3^Department of Psychiatry and Psychotherapy, University Medical Center, Johannes Gutenberg-University, Mainz, Germany; ^4^Research Center for Immunotherapy (FZI), Johannes Gutenberg-University, Mainz, Germany

**Keywords:** traumatic brain injury, ADAM10 (a disintegrin and metalloprotease 10), GI254023X, neuroprotection, neuroinflammation, axonal injury

## Abstract

The α-secretase A disintegrin and metalloprotease 10 (ADAM10) regulates various physiological and pathophysiological processes. Despite its broad functional implications during development, plasticity, and disease, no pharmacological approaches to inhibit ADAM10 in acute brain injury have been reported. Here, we examined the effects of the ADAM10 inhibitor GI254023X on the neurological and histopathological outcome after experimental traumatic brain injury (TBI). C57BL/6N mice were subjected to the controlled cortical impact (CCI) model of TBI or sham procedure and received GI254023X or vehicle during the acute phase of injury (*n* = 40, 100 mg/kg, 25% DMSO, 0.1 M Na_2_CO_3_, intraperitoneal, 30 min and 24 h after TBI). GI254023X treatment did not improve neurological deficits from 1 to 7 days post-injury (dpi) but animals treated with GI254023X exhibited smaller brain lesions compared to vehicle treatment. Determination of brain mRNA expression by quantitative PCR showed that TBI-induced up-regulation of *Adam10* and *Adam17* was not influenced by GI254023X but the up-regulation of the matrix metalloproteinase genes *Mmp2* and *Mmp9* was attenuated. GI254023X treatment further increased the T cell marker *Cd247* but did not affect blood brain barrier integrity, as assessed by Occludin mRNA expression and IgG brain extravasation. However, in agreement with neuroprotective effects of ADAM10 inhibition, GI254023X treatment attenuated axonal injury, as indicated by decreased generation of spectrin breakdown products (SBDPs) and decreased immunostaining using anti-non-phosphorylated neurofilament (SMI-32). Interestingly, reduced axonal injury in GI254023X-treated animals coincided with subtle mRNA dysregulation in the glutamate receptor subunit genes *Gria1* and *Grin2b*. Quantitative PCR also revealed that GI254023X mitigated up-regulation of the pro-inflammatory markers *Il6*, *Tnfa*, and *Lcn2* but not the up-regulation of the pan-microglia marker *Aif1*, the M2 microglia marker *Arg1* and the reactive astrocyte marker *Gfap*. Taken together, the ADAM10 inhibitor GI254023X attenuates brain tissue loss, axonal injury and pro-inflammatory gene expression in the CCI model of TBI. These results suggest that ADAM10 may represent a therapeutic target in the acute phase of TBI.

## Introduction

Traumatic brain injury (TBI) is a leading cause of death and disability and 69 million individuals worldwide are estimated to sustain a TBI each year (Dewan et al., 2018). The initial primary injury leads to irreversible tissue damage and triggers a plethora of secondary processes, such as defective cerebral perfusion and autoregulation, breakdown of the BBB, early formation of brain edema, mitochondrial dysfunction, oxidative stress, and other processes cumulating in the induction of neuronal cell death (Werner and Engelhard, 2007; Schäfer et al., 2014; Abdul-Muneer et al., 2015; Akamatsu and Hanafy, 2020). Subsequently, the activation of brain resident glia and infiltration of peripheral immune cells toward injury sites generates a highly inflammatory environment and oxidative stress, which is unfavorable for neuronal survival (Schäfer and Tegeder, 2018; Morganti-Kossmann et al., 2019).

Unfortunately, the pathophysiology of TBI is still incompletely understood and decades of animal research providing pharmacological targets for early therapeutic interventions have not been yet translated to the clinic. Given the complexity of TBI, the targeting of multiple secondary injury pathways has been proposed to better support neuronal survival and brain repair than targeting single factors (Loane and Faden, 2010; Hummel et al., 2020). However, also the targeting of single factors can have profound implications when they are at the forefront of regulatory processes such as enzymes with a variety of substrates executing different biological effects.

One such potential target is the metalloproteinase ADAM10, which can cleave almost 100 different substrates, including Notch family members, amyloid precursor protein (APP), cell adhesion molecules such as cadherin and members of the L1 family of cell adhesion molecules, the IL-6 cytokine receptor and membrane-bound chemokines (Wetzel et al., 2017; Hsia et al., 2019). ADAM10 has been best characterized in the context of early neural development (Jorissen et al., 2010), synapse function (Prox et al., 2013) and the non-amyloid pathway which prevents accumulation of neurotoxic Aβ peptides—hallmarks of Alzheimer’s disease (AD)—and generates potentially neuroprotective APP α-fragments (Thornton et al., 2006; Claasen et al., 2009; Gralle et al., 2009). Furthermore, the proteolytic activity of ADAM10 plays an important role in immunity and several inflammatory pathologies (Dreymueller and Ludwig, 2017; Lambrecht et al., 2018) and its dysregulation is linked to various types of cancer such as breast cancer (Tsang et al., 2018), colon cancer (Gavert et al., 2007), and malignant pleural mesothelioma (Sépult et al., 2019).

In contrast, few studies have investigated the role of ADAM10 in acute brain injury. Using unilateral entorhinal cortex lesion (ECL) in rodents, ADAM10 mRNA and protein expression up-regulation was observed in the denervated dentate gyrus, presumably in astrocytes (Warren et al., 2012; Del Turco et al., 2014). ADAM10 increased within 7 days post-injury—during reactive astrogliosis, axonal sprouting, and reactive synaptogenesis—and evidence was provided that ADAM10-mediated cleavage of N-cadherin prevents synaptic reorganization and thereby interferes with functional recovery (Warren et al., 2012). Another study reported that suppression of ADAM10 expression by miR-144 increased Aβ peptide accumulation and aggravated cognitive impairments within 1 week following TBI (Sun et al., 2017). Along this line, ADAM10 up-regulation has been implicated in decreased Aβ production and synapse rescue mediated by the PKC activator bryostatin 1 in a model of mild TBI (Zohar et al., 2011). However, also deleterious effects of ADAM10 were inferred from experiments using transgenic mice, expressing a dominant-negative ADAM10 mutant (ADAM10-dn). These mice showed a decreased seizure score and less hippocampal neuronal damage following kainate administration (Clement et al., 2008). More recently, layer-specific upregulation of ADAM10 was demonstrated in cortical neurons and microglia after whisker lesioning. In this model of cortical synapse remodeling, GI254023X, a selective inhibitor of ADAM10 (Ludwig et al., 2005), prevented ADAM10-mediated cleavage of the fraktalkine receptor CX3CR1 and thereby microglia-mediated synapse elimination (Gunner et al., 2019). Together, these studies imply a role for ADAM10 in various models of acute brain injury.

In this study, using controlled cortical impact (CCI) as a clinically relevant model of TBI, we examined the effects of GI254023X administration during the acute stage, at 30 min and 24 h post trauma, with respect to the neurological outcome and brain pathology at 7 days after injury (dpi). Combining behavioral assessment (immuno-) histology, immunohistochemistry, immunoblot, and mRNA expression analyses of various markers, we tested the hypothesis that pharmacological inhibition of ADAM10 has beneficial effects after experimental TBI.

## Materials and Methods

### Animals

All animal experiments were performed after approval by the animal Care and Ethics Committee of the Landesuntersuchungsamt Rheinland-Pfalz (protocol 23177-07/G13-01-018) observing the institutional guidelines of the Johannes Gutenberg University, Mainz, Germany. Adult male C57Bl/6N mice (8 weeks old) from Janvier (Germany) were investigated, weighing approximately 25 g. Animals were housed in a temperature-controlled environment (23 ± 1°C, 55 ± 5% relative humidity) with a 12 h light-dark circle and food and water *ad libitum*. A total of 40 mice was used in this study and randomly allocated to four experimental groups (sham: vehicle *n* = 9, GI254023X *n* = 7; CCI: vehicle *n* = 12, GI254023X *n* = 12). All experimenters, one performing the sham or CCI procedure and another one performing drug application, behavioral tests and tissue preparations, were blind to the treatment groups.

### Experimental Traumatic Brain Injury, Physiological Parameters, and Behavioral Analysis

The CCI model was used to induce an experimental brain injury as described before (Hummel et al., 2020). Briefly, after induction of anesthesia (isoflurane 4 Vol.%, maintenance 2.1 Vol.%) mice were attached to a stereotactic apparatus (Kopf Instruments) and a right parietal craniotomy was performed. CCI was induced according to the following parameters: impactor tip diameter: 3 mm, impact velocity: 6 m/s, impact duration: 200 ms, displacement: 1.5 mm). After the procedure, skull and skin were carefully closed and the animals transferred to a neonatal incubator (IC8000, Draeger, Luebeck, Germany) for approximately 1 h with controlled air temperature and ambient humidity. Sham animals were handled identically in terms of anesthesia and skin incision. Since craniotomy already leads to brain damage in our TBI model (Cole et al., 2011) only slight drilling on the exposed skull surface instead of craniotomy was performed. Body temperature was controlled by a feedback heating device and adjusted to 37°C intraoperatively. Physiological and technical parameters, including body weight, rectal and pericranial temperature as well as anesthesia time and duration of operation, were monitored before and/or during the operation (Supplementary Table 1). Behavioral tests were performed in a blinded and unbiased fashion 1 day before and 1 and 7 days after CCI using a neurological severity score (NSS). The NSS was modified from Tsenter et al. (2008) and assesses the severity of neurological impairments ranging from 0 (no impairment) to 12, as described (Hummel et al., 2020).

### Drug Treatment

The pharmacological ADAM10 inhibitor GI254023X was administered via intraperitoneal injection (100 mg/kg body weight, 30 min and 24 h after CCI). GI254023X was purchased from Aobious (CAS No.: 260264-93-5), and dissolved in 25% DMSO in 0.1 M Na_2_CO_3_. Control mice were handled identically but received vehicle solution (25% DMSO in 0.1 M Na_2_CO_3_). The anti-proteolytic activity of GI254023X was confirmed *in vitro* prior to *vivo* administration (Supplementary Figure 1).

### Brain Sectioning and Histology

After the observation period of 7 days, mice were sacrificed by cervical dislocation in deep anesthesia (4 vol% isoflurane). Brains were collected and frozen in dry powdered ice and stored at −80°C. Serial cryosections of 12 μm were prepared using a cryostat (HM 560 Cryo-Star, Thermo Fisher Scientific, Walldorf, Germany) and collected in 500 μm intervals on glass slides (Superfrost plus). Sections were collected from Bregma +3.14 to −4.36 mm. Tissues between the intervals were processed for protein and RNA extractions as described (Menzel et al., 2017; Krämer et al., 2019). Cresyl violet staining was performed with 16 consecutive sections per mouse brain, images were taken using a stereo microscope (Stemi 305, Zeiss, Oberkochen, Germany) and the brain lesion areas were determined using ZEN imaging software tools (Zeiss, RRID:SCR_013672) by investigators blind to the treatment groups. Brain lesion volume was calculated by summation of areas multiplied by the distance between sections. Data were expressed relative to the volume of the ipsilesional hemisphere.

### Immunohistochemistry and Immunoblotting

Analysis of immunohistochemical images for non-phosphorylated neurofilament was performed essentially as described (Menzel et al., 2017). Briefly, cryosections were fixed in 4% paraformaldehyde (PFA) in phosphate buffered saline (PBS) for 10 min, washed in PBS and incubated in blocking solution (5% goat serum, 0.5% bovine serum albumin, and 0.1% Triton-X-100, in PBS) for 1 h at room temperature. Primary antibodies were applied in blocking solution (Covance, mouse anti-non-phosphorylated neurofilament heavy chain, NF-H, clone SMI-32, 1:1,000, RRID:AB_509998) overnight at 4°C. Sections were then washed in PBS and incubated with an Alexa488-conjugated secondary anti-mouse antibodies (Life Technologies, Grand Island, NY, United States, 1:500, RRID:AB_2534069). Images were acquired using a confocal scanning microscope (LSM510, Zeiss) and identical filter and acquisition parameters. Analysis for non-phosphorylated neurofilament was performed using the ImageJ software (ImageJ, RRID:SCR_003070), appropriate threshold settings and the “Analyze Particle” plugin essentially as described (Menzel et al., 2017). All investigators were blinded to the treatment groups.

For immunoblotting, brain samples were collected during cryosectioning and homogenized in RIPA buffer and protein concentrations were determined by Lowry assay (BioRad). Immunoblotting and dot blot analysis were performed essentially as described (Hummel et al., 2020). Briefly, 30 μg of protein for each sample was resolved by 10% SDS-PAGE. Nitrocellulose membranes were washed in TBS containing 0.1% Tween, blocked with 2.5% non-fat milk and incubated with mouse anti-α-fodrin/αII-spectrin (Enzo Life Sciences, 1:750, RRID:AB_10554860), mouse anti-GAPDH (Acris Antibodies GmbH, 1:1,000, RRID:AB_1616730), and secondary infrared (IR) dye-conjugated antibodies (LI-COR; goat anti-mouse IgG IRDye 800, RRID:AB_10793856). For dot blot analysis, 10 μg of brain tissue protein (5 μg/μl, lysed in RIPA buffer) were dotted onto a nitrocellulose membrane, washed and incubated with goat anti-mouse IgG IRDye 800 (LI-COR, 1:10,000) for 1 h at room temperature. Protein band or protein dot densities were digitalized and quantified using the Odyssey SA imaging system and Odyssey CLx software (LI-COR biotechnology, RRID:SCR_014579).

### Gene Expression Analysis

Gene expression was quantified as outlined before (Hummel et al., 2020). Briefly, RNA was extracted from brain tissue and reverse-transcribed into cDNA using RNeasy Kit and QuantiScript Reverse Transcription Kit (Qiagen), respectively. Equal amounts of cDNA were amplified by qPCR using a LightCycler (Hoffmann-La Roche AG). All values were normalized to the reference gene (PPIA) and absolute quantification was performed using a target specific standard curve and LightCycler Software (Hoffmann-La Roche AG, RRID:SCR_012155) and obtained data were expressed relative to the corresponding sham condition. Primer sequences, annealing temperature, and amplification products lengths are listed in Table 1.

**TABLE 1:** Oligonucleotide primers.

qPCR assay (gene name, protein name, amplicon size and annealing temperature)	Oligonucleotide sequences 5′–3′	Gene accession number
Adam10 (ADAM10, 151 bp, 58°C)	fw-TCATGGGTCTGTCATTGATGGA rev-TCAAAAACGGAGTGATCTGCAC	NM_007399
Adam17 (ADAM17, 100 bp, 58°C)	fw-GGATCTACAGTCTGCGACACA rev-TGAAAAGCGTTCGGTACTTGAT	NM_009615
Aif1 (Iba1, 144 bp, 58°C)	fw-ATC AAC AAG CAA TTC CTC GAT GA rev-CAG CAT TCG CTT CAA GGA CAT A	NM_019467
Arg1 (Arginase 1, 185 bp, 58°C)	fw-CTCCAAGCCAAAGTCCTTAGAG rev-AGGAGCTGTCATTAGGGACATC	NM_007482
Camk2a (CAMK2A, 109 bp, 58°C)	fw-TATCCGCATCACTCAGTACCTG rev-GAAGTGGACGATCTGCCATTT	NM_009792.3
Cd247 (CD3zeta, 150 bp, 58°C)	fw-CTGCTACTTGCTAGATGGAATCC rev-TCTCTTCGCCCTAGATTGAGC	NM_001113391
Gfap (GFAP, 120 bp, 58°C)	fw-CGG AGA CGC ATC ACC TCT G rev- TGG AGG AGT CATTCG AGA CAA	NM_001131020
Gria1 (AMPAR1, 152 bp, 58°C)	fw-ATGTGGAAGCAAGGACTCCG rev-TCGACTCGCTACGGGATTTG	NM_001113325
Grin2b (NMDAR2B, 222 bp, 58°C)	fw-AAGCTGCCTTTCTATCCCCG rev-GTGGTCATTCCCAAAGCGTC	NM_008171
Il1b (IL-1b, 348 bp, 58°C)	fw-GTG CTG TCG GAC CCA TAT GAG rev-CAG GAA GAC AGG CTT GTG CTC	NM_008361
Il6 (IL-6, 471 bp, 55°C)	Cy5–TGCTCTCCTAACAGATAAGCTGGAGTCAC–PH fw(ex2,3)-TCGTGGAAATGAGAAAAGAGTTG FL-CATAAAATAGTCCTTCCTACCCCAATTTCC-FL Rev(ex5,6)-TATGCTTAGGCATAACG ACTAG	NM_031168
Lcn2 (Lipocalin-2, 239 bp, 58°C)	fw-TGGCCCTGAGTGTCATGTG rev-CTCTTGTAGCTCATAGATGGTGC	NM_008491
Mmp2 (MMP2, 140 bp, 58°C)	fw-ACCTGAACACTTTCTATGGCTG rev-CTTCCGCATGGTCTCGATG	NM_008610
Mmp9 (MMP9, 106 bp, 58°C)	fw-AAGTCTCAGAAGGTGGAT rev-AATAGGCTTTGTCTTGGTA	NM_013599
Ocln (Occludin, 268 bp, 55°C)	Cy5-AGATGCCAGTTGCGGGAGAA fw-GCAAATTATCGCACATCAAGAG FL-GGAGATTATGACAGACGGAAACCTTAG rev-TGTTCAGCCCAGTCAATTATC	NM_008756
Ppia (cyclophilin A, 146 bp, 58°C)	fw-GCGTCTSCTTCGAGCTGTT rev-RAA GTC ACC CTG GCA	NM_008907
Tnfa (TNFα, 212 bp, 58°C)	fw-TCT CAG TTC TAT GGC CC rev-GGG AGT AGA CAA GGT ACA AC	NM_013693

### Statistical Analysis

All analyses were performed using GraphPad Prism^®^ (RRID:SCR_002798). Data distribution was analyzed using the Shapiro-Wilk-test. Dependent on data distribution, differences in pairwise comparisons were calculated by the Student’s *t*-test or the Mann-Whitney-U test. Multiple groups over different time points (body weight) were analyzed by two-way ANOVA, whereas non-parametrically distributed groups (NSS) were analyzed via Kruskal-Wallis test separately for each time point followed by Dunn’s multiple comparison test. If not otherwise stated, all other data were expressed relative to the corresponding sham condition. If applicable, data outliers were identified using ROUT’s test and removed as outlined in figure legends. Values from individual mice are shown as mean ± SEM, *^∗^p* < 0.05, ^∗∗^*p* < 0.01, ^∗∗∗^*p* < 0.001, ^****^p < 0.0001.

## Results

### GI254023X Does Not Attenuate Neurological Deficits but Brain Tissue Loss After TBI

**FIGURE 1:**
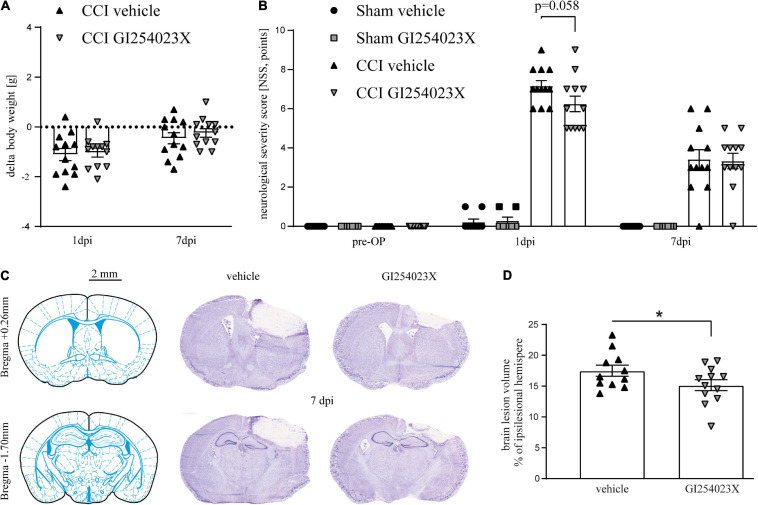
GI254023X does not attenuate neurological deficits but brain tissue loss after TBI. **(A)** Loss of body weight at 1 and 7 dpi compared to 1 day pre-OP in vehicle- or GI254023X-treated CCI mice. The dotted line refers to the pre-operation body weight. No differences were observed between vehicle and GI254023X treated mice as tested by two-way ANOVA. **(B)** Evaluation of neurological deficits using a NSS (0 = no impairment, 12 = maximal impairment). Animals were tested at the day before surgery (pre-OP), at 1 and 7 dpi. No differences were observed between vehicle and GI254023X treated mice as tested by Kruskal-Wallis test for each time point. **(C)** Schemes showing Bregma levels at +0.26 and −1.7 mm (Franklin and Paxinos Mouse Atlas 3rd edition) and cresyl violet stained coronal brain cryosections. Scale bar: 2 mm. **(D)** Brain lesion volumetry calculated from 16 consecutive sections at 7 dpi showing smaller lesions in GI254023X compared to vehicle treated mice as tested by Student’s *t*-test (*p < 0.05). Data are expressed as lesion volume in% of the ipsilesional hemisphere. One outlier was removed after identification by ROUT’s test in **(D)**. Values from individual animals and mean ± SEM are shown.

To study the influence of ADAM10 inhibition in acute brain injury, we used the CCI model of TBI which allows induction of highly reproducible and region-specific cortical injury (Smith et al., 1995). Mice were subjected to CCI or sham procedure. Body weight, rectal and pericranial temperature, as well as anesthesia and surgery duration of the procedure were within the expected range, before and during the procedure, respectively, and did not differ between the groups (Supplementary Table 1). GI254023X (100 mg/kg, i.p.) or vehicle were administered during the acute phase of TBI, at 30 min and 24 h after trauma. TBI led to a minor weight loss within the first 24 h after induction, which was almost restored at 7 dpi both in vehicle and GI254023X treated mice (Figure 1A). To assess neurological deficits and recovery after TBI, we used a modified 12-point neurological severity score (NSS) comprising motor ability, alertness, balance, and general behavior. Pronounced neurological deficits were evident at 1 dpi in CCI compared to sham groups (Figure 1B). At 7 dpi, the neurological deficits were decreased in the CCI groups compared to 1 dpi. However, the NSS did not differ between GI254023X- and vehicle-treated animals at 1 or 7 dpi (Figure 1B). To determine the structural brain damage at 7 dpi, brains were cut to coronal slices starting at Bregma + 3.14 mm and were stained with cresyl violet (Figure 1C). Next, the histopathological brain damage was determined by lesion volumetry (Figure 1D). This analysis revealed significantly smaller brain lesions in GI254023X-treated as compared to vehicle-treated mice. Hence, the posttraumatic administration of the ADAM10 inhibitor GI254023X does not attenuate neurological deficits but reduces brain tissue loss after TBI.

### GI254023X Influences Matrix Metalloproteinase Gene Expression but Not Overall BBB Integrity After TBI

**FIGURE 2:**
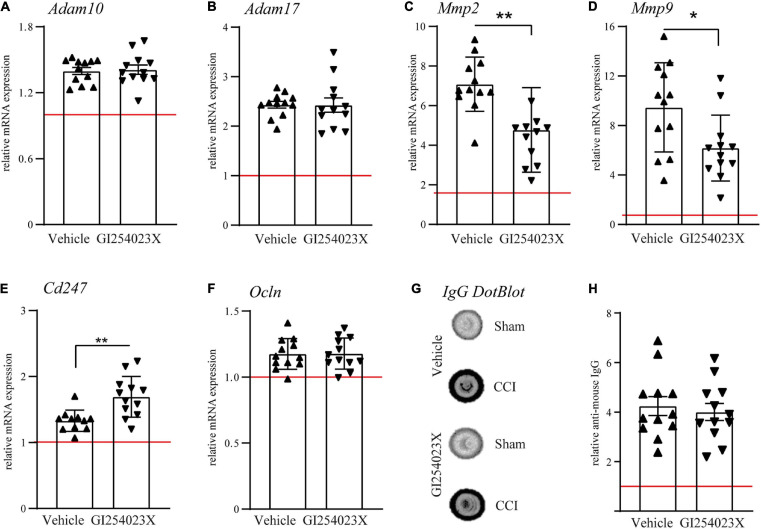
GI254023X influences matrix metalloproteinase gene expression but not overall BBB integrity after TBI. **(A–F)** Histograms showing relative mRNA expression of ADAM10, ADAM17, MMP2, MMP9, CD3 CD3zeta (*Cd247*), and Occludin (*Ocln)* at 7 dpi in ipsilesional brain tissue samples. **(G)** Representative anti-IgG Dot-blot using 7 dpi protein lysates from ipsilesional brain tissue. **(H)** Densitometric quantification of IgG dot blots as a proxy of BBB damage. mRNA expression values were normalized to the reference gene *Ppia* (cyclophilin A) and data are shown relative to the corresponding sham group (red line = sham). *P*-values were calculated by Student’s *t*-test or Mann-Whitney test (*p < 0.05, **p < 0.01). One outlier was removed after identification by ROUT’s test in **(E)**. Values from individual animals and mean ± SEM are shown.

To elucidate the effect of the ADAM10 inhibitor on the gene expression of metalloproteases, ipsilesional brain samples were investigated by qPCR at 7 dpi. Initially, mRNA expression levels of ADAM10 and its close homolog ADAM17 were examined. Both genes appeared up-regulated after TBI but no differences were observed between vehicle and GI254023X treatment (Figures 2A,B). Next, we examined expression regulation of the matrix metalloproteinase genes *Mmp2* and *Mmp9*, which have been associated with BBB damage and neuronal cell death after TBI (Sifringer et al., 2007; Vajtr et al., 2009). Both genes appeared up-regulated in response to TBI but their expressions were attenuated by GI254023X treatment (Figures 2C,D). Previous findings on the role of ADAM10 on T cell migration (Schulz et al., 2008) prompted us to determine gene expression of the CD3 zeta subunit (Cd247) as a pan-T cell marker. Interestingly, the up-regulation of this gene was more pronounced in GI254023X-treated as compared to vehicle-treated animals (Figure 2E). Data on the mRNA expression of MMP2, MMP9, and Cd247 suggested alterations in BBB integrity. Therefore, we assessed BBB integrity by determining mRNA levels of the tight junction marker Occludin (Figure 2F) and the extravasation of Immunoglobulin G (IgG) as a proxy of BBB permeability (Figures 2G,H). However, we did not find differences for these markers suggesting that GI254023X did not influence overall BBB integrity after TBI.

### GI254023X Reduces Axonal Injury and Alters the Gene Expression of Glutamate Receptor Subunits After TBI

**FIGURE 3:**
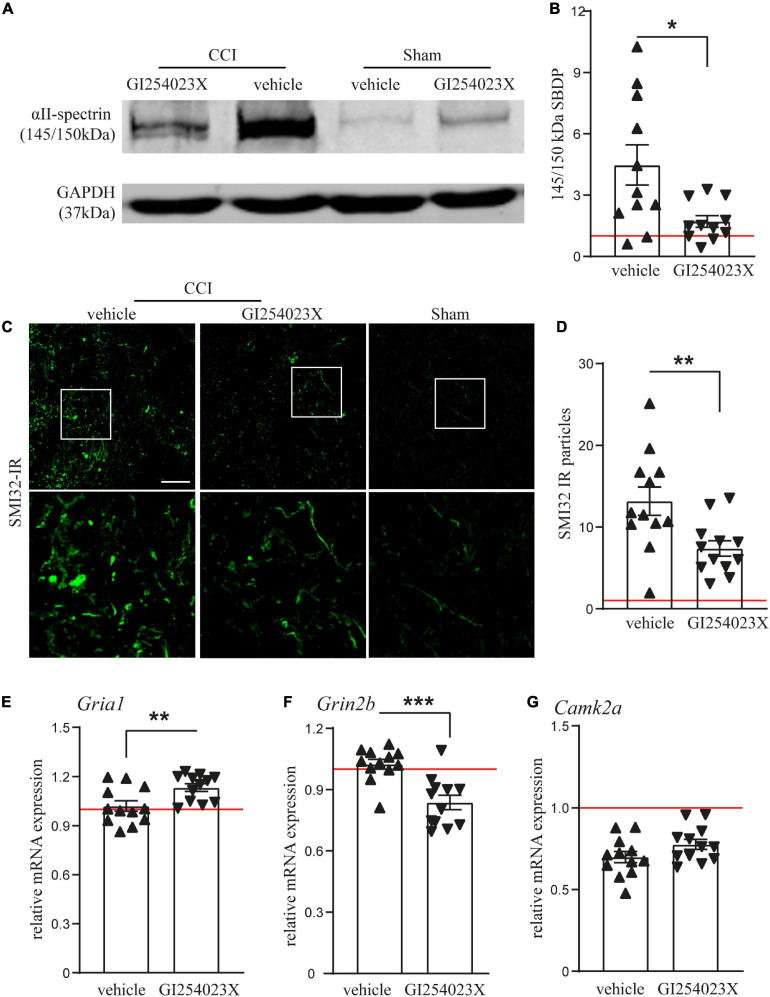
GI254023X reduces axonal injury and alters the gene expression of glutamate receptor subunits after TBI. **(A)** Representative western blot showing TBI-induced generation of spectrin breakdown products (SBDPs, 145–150 kDa) at 7 dpi in ipsilesional brain tissue samples. **(B)** Semi-quantitative densitometry of protein bands revealed reduced levels of SBDPs in GI254023X-treated animals compared to vehicle treatment (*n* = 11 each). **(C)** Immunofluorescence images of cryosections for non-phosphorylated Neurofilament-H (SMI-32) at 7 dpi at perilesional sites indicate increased axonal injury at 7 dpi in CCI animals compared to sham. **(D)** Quantitative assessment of SMI-32 immunoreactive (IR) particles at 7 dpi showing decreased numbers of SMI-32-IR particles in GI254023X- compared to vehicle-treated animals (*n* = 12 each). **(E–G)** Histograms showing relative mRNA expression of AMPAR1 (*Gria1*), NMDAR2B (*Grin2b)*, and CamK2A (*Camk2a*) at 7 dpi in ipsilesional brain tissue samples. Expression values were normalized to the reference *Ppia* (cyclophilin A) and data from CCI animals (*n* = 12 each group) are shown relative to sham (red line = sham). Two outliers were removed after identification by ROUT’s test in **(B)**. Values from individual animals and mean ± SEM are shown. Differences between GI254023X- and vehicle-treated animals were calculated by Student’s *t*-test (*p < 0.05, **p < 0.01, ***p < 0.001).

Our results showing smaller brain lesions in GI254023X-treated mice after TBI, prompted us to examine issues of brain homeostasis at 7 dpi. Therefore, we analyzed αII-spectrin breakdown products (SBDPs), which serve as indicators for disturbed neuronal Ca^2+^ homeostasis, axonal injury and degeneration after TBI (Gölz et al., 2019). Immunoblot analysis of protein lysates from ipsilesional brain tissues and corresponding samples from sham mice revealed a marked increase of the 145/150 kDa SBDPs (Figure 3A), known to result from increased caspase and calpain activity (Zhang et al., 2009). There was a 4.5 fold increase of SBDPs in brain lysates from vehicle treated mice but only a 1.7 fold increase in the corresponding brain lysates from GI254023X treated mice (Figure 3B). To further assess trauma-induced axonal injury at perilesional sites, we immunostained brain cryosections with the SMI-32 antibody clone specific to non-phosphorylated neurofilament (Figure 3C). The number of SMI-32 immunoreactive particles was lower in mice treated with GI254023X than in vehicle-treated mice (Figure 3D). As glutamate receptors play an important role in regulating intracellular calcium levels (Krishnamurthy and Laskowitz, 2016) and trigger neurotoxicity after TBI (Carvajal et al., 2016), we performed mRNA expression analyses of ipsilesonal brain tissue by quantitative PCR for the glutamate ionotropic receptor AMPA type subunit 1 (*Gria1*), the glutamate ionotropic receptor NMDA subunit 2B (*Grin2b*), and the Calcium/calmodulin-dependent protein kinase type II alpha chain (*Camk2a*) (Figures 3E–G). We found that GI254023X treatment increased the expression of *Gria1* but lowers the expression of *Grin2b* compared to vehicle-treated animals at 7 dpi (Figures 3E,F). No effects of GI254023X were observed on the expression of *Camk2a* (Figure 3G). Together, these results show that GI254023X reduces axonal injury and alters the gene expression of glutamate receptor subunits after TBI.

### GI254023X Attenuates Pro-inflammatory Gene Expression After TBI

**FIGURE 4:**
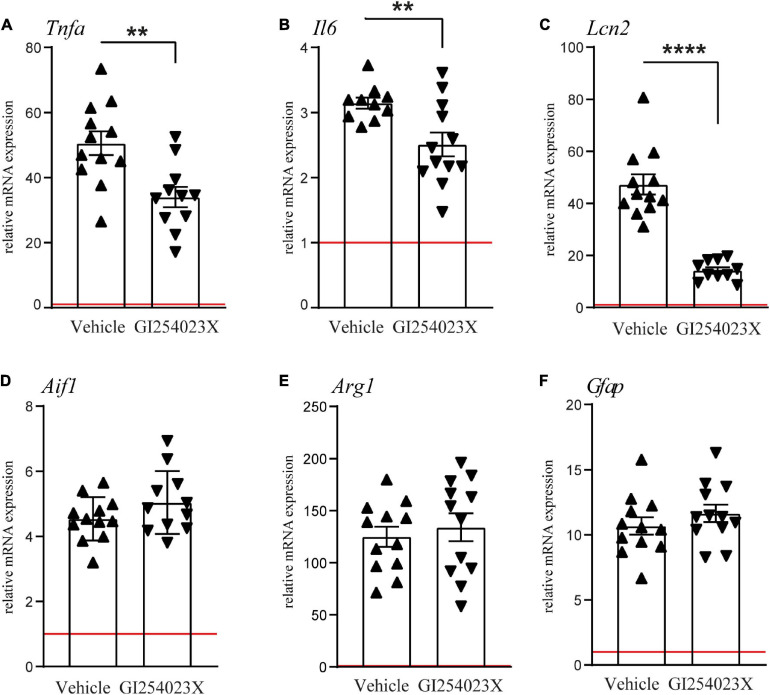
GI254023X attenuates pro-inflammatory gene expression after TBI. **(A–F)** Histograms showing relative mRNA expression of TNFa (*Tnfa*), IL-6 (*Il6*), LCN2 (*Lcn2*), Iba1 (*Aif1*), Arginase1 (*Arg1*), and GFAP (*Gfap*) at 7 dpi in ipsilesional brain tissue samples. Expression values were normalized to the reference gene *Ppia* and data are shown relative to sham (red line = sham). Outliers were removed from GI254023X- or vehicle-treated CCI groups after identification by ROUT’s test. Values from individual animals and mean ± SEM are shown. *P*-values were calculated by Student’s *t*-test **(A–C)** (**p < 0.01, ****p < 0.0001).

As ADAM10 activity regulates a variety of inflammatory processes, we examined mRNA expressions of the pro-inflammatory cytokines TNFα and IL-6 and the acute phase factor LCN2 along with markers for microglia/macrophages and/or reactive astrocytes (Figures 4A–F). TNFα and IL-6 mRNA expression levels were increased in response to TBI at 7 dpi but their up-regulation was attenuated in GI254023X-treated animals (Figures 4A,B). Similarly, LCN2 mRNA expression, which has been associated with astrocyte-mediated neurotoxicity (Bi et al., 2013), was induced by TBI but GI254023X attenuated its up-regulation (Figure 4C). However, no differences were found in the mRNA expression of the pan-microglia marker Aif1 or the M2 microglia marker Arg1 (Figures 4D,E). Also, the reactive astrocyte marker GFAP was induced by TBI but not different between GI254023X and vehicle-treated animals (Figure 4F). Together, these results show that GI254023X attenuates pro-inflammatory gene expression after TBI.

## Discussion

We here report that the ADAM10 inhibitor GI254023X attenuates brain tissue loss, axonal injury and pro-inflammatory gene expression in the murine CCI model of TBI. GI254023X is a selective and potent inhibitor of ADAM10 that chelates the Zn^2+^ of the protease active site (Ludwig et al., 2005). A 100-fold selectivity of GI254023X was reported for the α-secretase ADAM10 over ADAM17 and several studies confirmed its selectivity (Pruessmeyer et al., 2014; Brummer et al., 2018; Vezzoli et al., 2019). GI254023X has been applied in various animal disease models. Beneficial effects were found in a mouse model of muscle injury and regeneration in which both genetic inhibition of ADAM10 and its pharmacological inhibition by GI254023X accelerated regeneration (Mizuno et al., 2018). In a bacterial sepsis model, GI254023X reduced vascular injury, vascular endothelial (VE) cadherin cleavage and concomitant loss of endothelial barrier function (Powers et al., 2012). Other studies using administration of GI254023X focused on mouse models of chronic neurodegenerative diseases such as Huntington disease (Vezzoli et al., 2019). It has been concluded that the inhibition of the proteolytic activity of ADAM10 by GI254023X has neuroprotective potential in this model. Interestingly, even in the context of AD, where beneficial effects of ADAM10 or its activation were shown by many laboratories in AD animal models (Postina et al., 2004; Tippmann et al., 2009; Zhang et al., 2013; Corbett et al., 2015; Meng et al., 2017) administration of GI254023X showed neuroprotective potential (Shackleton et al., 2016). Altogether, there is consensus on beneficial effects of GI254023X in various disease conditions and our study extends these findings in an animal model of TBI.

Up to date, ADAM10 inhibition by GI254023X has not been reported in the context of TBI or stroke, which share many pathophysiological features (Bramlett and Dietrich, 2004; Schäfer et al., 2014). In the present study we applied GI254023X at 30 min and 24 h after TBI which corresponds to the acute phase (Toshkezi et al., 2018). Previous studies reported upregulation of ADAM10 from 2 to 15 days after ECL (Warren et al., 2012) or 7 days after ECL (Del Turco et al., 2014). Our mRNA expression data suggest that ADAM10 expression is slightly up-regulated at 7 dpi, the time-point of histopathological examination. It has been proposed that ADAM10 plays a plasticity-enhancing and neuroprotective role during the first phase following injury. It shapes the extracellular environment for sprouting fibers, clears synaptic sites, and liberates neuroprotective APP fragments (Endres and Deller, 2017). Our results showing smaller lesions and reduced axonal injury in GI254023X-treated mice at 7 dpi contradict earlier assumptions that ADAM10 plays a neuroprotective role during the first phase following cerebral injury (Endres and Deller, 2017). It is possible, however, that the inherent differences between the ECL model and the CCI model of TBI used in this study, particularly in terms of the induction and localization of injury, may have an impact on whether inhibition or activation of ADAM10 exerts neuroprotective effects. Our results showing reduced αII-spectrin cleavage and less SMI32-immunoreactivity in the injured hemisphere indicate neuroprotective effects of GI254023X. These effects coincided with downregulation of *Gria1* and upregulation of *Grin2b*, coding for the AMPA receptor subunit 1 (AMPAR1) and the NMDA receptor subunit 2B (NR2B), respectively. The downregulation of *Grin2b* in GI254023X-treated animals is intriguing as glutamate binding to extrasynaptic NR2B subunit triggers neurotoxicity (Carvajal et al., 2016). At the present, we can only speculate that expression changes of *Gria1* and *Grin2b* contribute to the beneficial effects of ADAM10 inhibition by GI254023X. However, the neuroprotective effects of GI254023X observed in our study are in agreement with previous work demonstrating that transgenic mice expressing a dominant-negative ADAM10 mutant (ADAM10-dn) showed less neuronal cell death and neuroinflammation after kainate injection than wild-type mice, which indicates beneficial effects of ADAM10 inhibition in context with neurodegeneration (Clement et al., 2008).

Interestingly, ADAM10 has been ascribed a pro-inflammatory role in the early phase of acute lung inflammation through improved alveolar recruitment of leukocytes (Pruessmeyer et al., 2014). ADAM10 also enhances transendothelial migration of T cells (Schulz et al., 2008) which was inhibited by GI254023X. In addition, GI254023X increased T cells’ killing capacity and increased the number of T cells undergoing activation-induced cell death (Schulte et al., 2007). These findings suggest that GI254023X may have anti-inflammatory effects via attenuation of T cells infiltration into the lesioned brain. As compared to vehicle-treated mice, we here found in GI254023X treated mice an increased Cd247 gene expression (coding for CD3zeta) at 7 dpi in the lesioned brain, which we have earlier shown to correlate with increased T cell infiltration (Krämer et al., 2019). However, the previous observations did not support a critical role of T cells in brain damage progression probably due to their low numbers in the brain parenchyma (Krämer et al., 2019). It appears therefore unlikely that T cells counteract or contribute to the ameliorative effects of GI254023X treatment on brain tissue loss and axonal injury in the present study.

An important finding of our study is that GI254023X attenuated the mRNA expression of the pro-inflammatory cytokines TNFα and IL6 and the acute phase factor LCN2. These factors are associated with worse outcomes in TBI and have been considered as therapeutic targets (Tuttolomondo et al., 2014; Rodney et al., 2018). TNFα and IL6 are important mediators of inflammation and cause neuronal cell death and neurological dysfunction but this may also trigger reparative processes (Lenzlinger et al., 2001). LCN2 gene expression is increased early after TBI in mice (Huang et al., 2016) and represents a potent neurotoxic factor (Bi et al., 2013). To the best of our knowledge, there are no published data on a possible connection between LCN2 and ADAM10 but several studies support a regulatory role of ADAM10 in TNFα and IL-6 mediated actions. ADAM10 cleaves and thereby mediates the release of TNFα (Arduise et al., 2008) and has also been linked to the regulation of neural IL-6 levels after activation by aromatic retinoid acetritin (Dos Santos Guilherme et al., 2019). It has been further reported that TNFα-induced apoptosis can be regulated via an ADAM10-NF-κB feedback loop in a B cell lymphoma cell line (Yang et al., 2019). A similar feedback loop was shown for the ADAM family member ADAMTS-7, which upregulates TNF-α and conversely, TNF-α induced ADAMTS-7 through NF-κB signaling (Lai et al., 2014). It is therefore possible that the neuroprotective and anti-inflammatory effects of GI254023X treatment found in the present study are partially due to the interference with feedback loops involving ADAM10, TNFα, and NF-κB signaling. As mentioned before, neuronal cell death and neuroinflammation were also reduced in ADAM10-dn mice compared to wild-type mice following kainate-induced acute brain injury (Clement et al., 2008). Hence, future studies are needed to uncover the existence of feedback loops between pro-inflammatory cytokines and ADAM10 in TBI and other types of acute brain injuries.

To conclude, we here studied for the first time pharmacological inhibition of ADAM10 in experimental TBI. Our results showing beneficial effects on brain histopathology and inflammatory response to TBI suggest that ADAM10 may represent a therapeutic target in the acute phase of TBI. Further studies in the CCI model and other models of TBI are required to optimize treatment regimen and to investigate whether the beneficial effects of ADAM10 inhibition are restricted to the acute phase or can lead to sustained brain protection after TBI.

## Data Availability Statement

The raw data supporting the conclusions of this article will be made available by the authors, without undue reservation.

## Ethics Statement

The animal study was reviewed and approved by the Animal Care and Ethics Committee of the Landesuntersuchungsamt Rheinland-Pfalz.

## Author Contributions

DA, RH, MW, and CG performed the experiments and collected the data. MS and KE conceived the study. MS, KE, and RH designed the experiments and data analysis. MS, DA, and RH performed the data analysis and wrote the manuscript. All authors approved the final manuscript.

## Conflict of Interest

The authors declare that the research was conducted in the absence of any commercial or financial relationships that could be construed as a potential conflict of interest.
