# Supplementary material for: Pharmacologic Inhibition of ADAM10 Attenuates Brain Tissue Loss, Axonal Injury and Pro-inflammatory Gene Expression Following Traumatic Brain Injury in Mice
Source: Front Cell Dev Biol. 2021 Mar 15;9:661462. doi: 10.3389/fcell.2021.661462 (PMC8005610; doi:10.3389/fcell.2021.661462)
Supplement: Supplementary file 1 [file Presentation_1.pdf]

Supplementary Material

GI254023X inhibits proteolysis of the ADAM10 substrate L1CAM in vitro

ADAM10 has been identified to cleave almost 100 different substrates including ectodomain shedding of the cell adhesion molecule L1CAM (Hsia *et al.* 2019; Schäfer & Altevogt 2010). L1CAM is a type 1 transmembrane protein with a large extracellular part comprising six Ig-like domains followed by five FNIII-type domains (Schäfer & Altevogt 2010) (Supplementary Figure 1A). The ADAM10 cleavage site was mapped to a membrane-proximal site of L1CAM resulting in the release of the 200 kDa extracellular part of L1CAM, comprising the extracellular IgG-like and FNIII-type domains (Mechtersheimer *et al.* 2001) (Supplementary Figure 1B).

To confirm the biological activity of GI254023X, we transiently transfected HEK293 cells with pcDNA3-L1CAM and/or pcDNA3-ADAM10 expression vectors and further cultured the cells in the presence or absence of GI254023X. Briefly, HEK293 cells were were cultured in DMEM supplemented with 10% fetal calf serum and 1% penicillin/streptomycin according to standard protocols, and transiently transfected with the expression vectors pcDNA3-ADAM10-HA (Anders *et al.* 2001), pcDNA3-L1CAM-wild-type (WT) and/or pcDNA3-L1CAM-W635C (Marx *et al.* 2012) using jetPRIME (Polyplus Transfection) according to the manufacturer’s protocols. Cells were further cultivated for 24 h in the presence or absence of 20 µM GI254023X. Next, the cells were lysed in ice cold radio-immunoprecipitation assay (RIPA) buffer (50 mM Tris-HCl, pH 7.5, 150 mM NaCl, 1 mM EDTA, 1% NP40, 0.1% sodium dodecyl sulfate, and complete protease inhibitors (Roche, Cat. No. 11697498001). Cell culture supernatants were concentrated using a Speed-Vac-Concentrator (Univapo 100H). Cell lysates and supernatants were subjected to SDS-PAGE and blotted on nitrocellulose membranes and probed using rabbit anti-human L1CAM (dilution 1:1000) (Schäfer *et al.* 2010) followed by secondary infrared (IR) dye-conjugated antibody goat anti-rabbit IRDye680 (LI-COR, dilution: 1:10.000, RRID:AB_621841). Protein band densities were digitalized and quantified using the Odyssey SA imaging system and Odyssey CLx software (LI-COR biotechnology, RRID: SCR_014579).

The immunoblot analysis using antibodies specific to the extracellular FNIII-like domains of L1CAM showed that GI254023X reduced the release of a ~200 kDa fragment into culture supernatants of cells transfected with L1CAM alone (Supplementary Figure 1C), probably due to endogenous expression

of ADAM10 by HEK cells (Brummer *et al.* 2018). A similar reduction was found in cells overexpressing both L1CAM and ADAM10 whereas the amounts of membrane-bound L1CAM were not obviously altered in total cell lysates (Supplementary Figure 1C). Overexpression of the cell surface trafficking defective L1CAM-W635C mutant (Marx *et al.* 2012), either alone or together with ADAM10, further suggested that ADAM10 cleaves L1CAM at the cell surface and not inside the cell (Supplementary Figure 1C). Taken together, the in vitro experiments show that GI254023X inhibits proteolysis of the ADAM10 substrate L1CAM in vitro thereby confirming its anti-proteolytic activity.

Supplementary Figure 1: GI254023X inhibits proteolysis of the ADAM10 substrate L1CAM in vitro

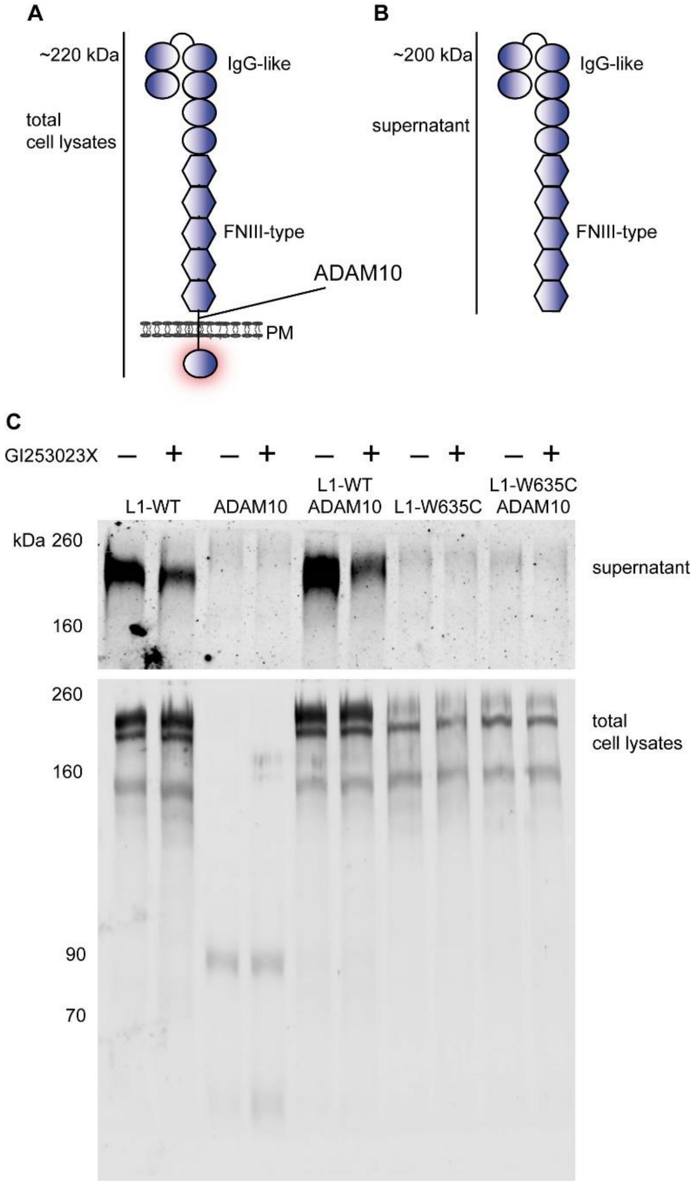

Supplementary references:

Anders, A., Gilbert, S., Garten, W., Postina, R. and Fahrenholz, F. (2001) Regulation of the alpha-secretase ADAM10 by its prodomain and proprotein convertases. *FASEB journal : official publication of the Federation of American Societies for Experimental Biology* **15,** 1837-1839.

Brummer, T., Pigoni, M., Rossello, A., Wang, H., Noy, P. J., Tomlinson, M. G., Blobel, C. P. and Lichtenthaler, S. F. (2018) The metalloprotease ADAM10 (a disintegrin and metalloprotease 10) undergoes rapid, postlysis autocatalytic degradation. *FASEB journal : official publication of the Federation of American Societies for Experimental Biology* **32,** 3560-3573.

Hsia, H.-E., Tüshaus, J., Brummer, T., Zheng, Y., Scilabra, S. D. and Lichtenthaler, S. F. (2019) Functions of 'A disintegrin and metalloproteases (ADAMs)' in the mammalian nervous system. *Cellular and Molecular Life Sciences* **76,** 3055-3081.

Marx, M., Diestel, S., Bozon, M. et al. (2012) Pathomechanistic characterization of two exonic L1CAM variants located in trans in an obligate carrier of X-linked hydrocephalus. *Neurogenetics* **13,** 49-59.

Mechtersheimer, S., Gutwein, P., Agmon-Levin, N. et al. (2001) Ectodomain shedding of L1 adhesion molecule promotes cell migration by autocrine binding to integrins. *The Journal of cell biology* **155,** 661-673.

Schäfer, M. K. and Altevogt, P. (2010) L1CAM malfunction in the nervous system and human carcinomas. *Cellular and molecular life sciences : CMLS* **67,** 2425-2437.

Schäfer, M. K., Nam, Y. C., Moumen, A. et al. (2010) L1 syndrome mutations impair neuronal L1 function at different levels by divergent mechanisms. *Neurobiology of disease* **40,** 222-237.

Supplementary Table 1

Table 1: Comparison of the physiological variables between vehicle and treatment groups

	Vehicle (25 % DMSO in 0.1 M Na_2CO_3)	GI254023X i.p. (100mg/ kg KG)
weight preoperative [g]	24.2±0.3	24.6±0.3
rectal temperature preoperative [°C]	38.0±0.2	37.8±0.2
rectal temperature intraoperative [°C]	37.0±0.04	37.0±0.1
pericranial temperature intraoperative [°C]	34.4±0.1	34.4±0.2
duration of operation [min]	17.7±0.6	18.3±0.8

i.p.: intraperitoneal, values are mean ± SEM, Body temperature was controlled by a feedback heating device and adjusted to 37° C intraoperatively. Physiological and technical parameters, including body weight, rectal and pericranial temperature as well as anesthesia time and duration of operation, were monitored before and/or during the operation.
